# Reduction of Orexin-A Is Associated With Anxiety and the Level of Depression of Male Methamphetamine Users During the Initial Withdrawal Period

**DOI:** 10.3389/fpsyt.2022.900135

**Published:** 2022-07-04

**Authors:** Lei Guo, Aqian Hu, Xiaoxi Zhao, Xiaojun Xiang

**Affiliations:** Department of Psychiatry, National Clinical Research Center for Mental Disorders, The Second Xiangya Hospital of Central South University, Changsha, China

**Keywords:** orexin-A (OXA), methamphetamine, withdrawal symptoms, anxiety, depression

## Abstract

**Background:**

Orexin has been linked to the regulation of reward and motivation in animals, but there have been few human studies to validate its regulatory effects. We aimed to determine how orexin-A levels changed during different stages of withdrawal, as well as the relationship between orexin-A levels and withdrawal symptoms in male METH users.

**Methods:**

This study included 76 METH users and 35 control participants. The METH users were divided into three groups: group 1 (abstinence within 1 week, *n* = 23), group 2 (abstinence between 1 week and 3 months, *n* = 38), and group 3 (abstinence over 3 months, *n* = 15). At baseline and 1 month of enrollment, the plasma orexin-A level was examined. To track the withdrawal symptoms, self-report questionnaires (anxiety, depression, craving, and sleep quality) were collected at two points.

**Results:**

The orexin-A levels of groups 1 (*p* < 0.001) and 2 (*p* < 0.001) were lower than that of the controls at baseline but not group 3. One month later, the orexin-A levels of group 2 (*p* < 0.05) significantly increased, while no significant changes in those of groups 1 and 3 were observed. Furthermore, the orexin-A levels of group 1 were positively linked with depression (*p* < 0.01) and anxiety (*p* < 0.01) at baseline.

**Conclusions:**

The decrease in orexin-A levels was only transitory during the initial abstinence phase, and it was eventually restored near to normal with continued abstinence among the male METH users. Furthermore, a lower concentration of orexin-A may serve as a risk factor for negative emotions during METH withdrawal.

## Introduction

Methamphetamine (METH) has resurfaced as a threat to global public health in the aftermath of the opioid crisis. METH use, as well as its associated mortality rate, has increased in the United States over the last 10 years (1); METH was the most often used and circulated illegal drug in China in 2019 (2). Anxiety, depression, disrupted sleep, cravings, and psychiatric problems are all withdrawal symptoms of prolonged METH exposure, and they are all linked to high rates of relapse (3). However, current knowledge of the complex neurobiological changes associated with METH use is in its infancy.

Orexin, also known as hypocretin, is a type of neuropeptide with two forms, which are orexin-A (OXA) and orexin-B (OXB). It is expressed exclusively in neurons in the lateral hypothalamus (LH), a brain region involved in reward and motivation, and is widely distributed throughout the brain, including the reward-associated key brain regions including the ventral tegmental area (VTA) and nucleus accumbens (4, 5). Orexin was first linked to feeding behavior (6) and sleep-wakefulness (7), but it is now obvious that it is part of the reward system and is involved in numerous stages of substance addiction (8). Orexin neurons can send direct signals to VTA dopamine (DA) neurons and cause presynaptic glutamate release, which facilitates the activation of VTA DA cells (9). Substance exposure increases excitatory inputs to VTA dopamine neurons in a similar way and blocking orexin-1 receptor signaling in the VTA inhibits the locomotor enhancement induced by repeated cocaine exposure, implying that orexin neurons are required for substance-induced upregulation of DA output (9, 10). In animal models, blocking orexin signaling reduces various addictive behaviors and prevents the relapse of drug use (11). Therefore, regulating orexin may be promising for managing cravings and reducing the risk of relapse of substance addiction (12). The National Institute on Drug Abuse has made efforts to investigate the function of orexin receptor antagonists and has designated orexin receptor antagonists as a top priority for the development of new treatments for substance abuse (13).

Previous studies of laboratory animals found that acute or chronic METH administration enhanced Fos expression in orexin neurons (14, 15), while orexin receptor antagonists inhibited METH-induced Conditioned Place Preference expression (16). The significance of orexin in METH users is unknown; however, it is fair to assume that the orexinergic system plays a critical role in METH addiction. One study showed decreased OXA concentrations in METH users during the drug withdrawal period (17). Another study found that their OXA concentrations increased during the acute withdrawal phase and decreased during the subacute withdrawal phase in people exposed to METH (18). Differences in METH use and withdrawal length may be attributed to the different findings. Furthermore, the relationship between orexin concentrations and withdrawal symptoms during METH cessation has not been established. In this study, we assessed the concentrations of OXA in male METH users during various withdrawal phases, as well as their relationship with withdrawal symptoms.

## Materials and Methods

### Participants

In this study, 76 male METH users were recruited from the Kangda Mental Health Center in the Hunan Province. The participants in the study met the diagnostic criteria for METH use disorder in the fifth edition of the Diagnostic and Statistical Manual of Mental Disorders (DSM-5) and had urine screening on the first day of enrollment. Individuals with a history of other substance abuse except nicotine were excluded. There was no history of severe physical sickness, psychological illness, or neurological illness among the participants. For at least 3 months before the trial, none of the participants had taken any psychiatric medicines, including antipsychotics and mood stabilizers. All the participants were in-patients who stayed in the hospital for the duration of the trial, during which medications were available. All the participants provided informed consent and completed the questionnaires. On day 1 and month 1 of enrolment, the plasma OXA concentrations and clinically significant withdrawal symptoms were assessed twice.

Thirty-five physically and mentally healthy and age-matched male adults from the Health Examination of Xiangya Second Hospital of Central South University were assessed for OXA concentrations for comparison with those of METH users. Participants completed a blood screening at enrollment, and those whose blood routine and biochemical indicators were significantly outside of the normal range were excluded. Individuals with a history of abuse of any substance, except nicotine, were excluded from the study. All healthy controls were free of any mental condition or medical disease.

### Data Collection and Clinical Evaluation

A total of 76 METH users and 35 controls were enrolled in the METH and healthy control groups, respectively. The METH users were allocated to three groups: group 1: abstinence for <1 week and a positive urine test for METH at enrollment; group 2: abstinence for between 1 week to 3 months; and group 3: abstinence for more than 3 months. The baseline sociodemographic data, including age, smoking habit, marital status, educational attainment, employment status, and body mass index (BMI), of the participants were collected using a self-made demographic survey questionnaire. Clinical data, such as the length of METH usage and medications during hospitalization, were also obtained. For METH users, baseline and 1-month follow-up interviews were conducted face-to-face. The 17-item Hamilton Depression Scale and 14-item Hamilton Anxiety Scale (19) were used to assess the severity of withdrawal-related negative effects including anxiety and depression. The Pittsburgh Sleep Quality Index (20) was used to assess sleep quality, and the Visual Analog Scale (on a scale of 1–10, the higher the score, the worse the craving) was used to assess METH craving.

### Blood and Sampling Collection and Determination

Compared to orexin-B, OXA can penetrate the blood-brain barrier passively, making it a convenient and trustworthy indicator (21). Given the influence of circadian rhythms on the stability of orexin concentrations, all blood samples were taken before breakfast between 7 and 8 a.m. On day 1 and month 1 of enrollment, we collected 3–5 milliliter blood samples using anticoagulant tubes following a fasting period of 8–10 h. The plasma OXA were separated using a centrifuge at 3,000 rpm for 8 min before being stored at −80 degrees until analysis. Wuhan Cloud-Clone Corp, China, identified and evaluated the plasma OXA using an enzyme-linked immunosorbent assay (Orexin-A kit: CEA607Hu 96T), following the manufacturer's instructions carefully and faithfully. The fluorescence intensities of the 96 well-microplates were read by an assay reader after the assays were completed. Standard curves were established with various concentrations of standard solutions, and the orexin-A concentrations in each sample were calculated by interpolation method. Orexin-A has a detection range of 12.35–1,000 pg/ml.

### Statistical Analysis

All data are presented as mean and standard of deviation. Before analysis, all continuous variables were subjected to normality tests. Pearson's chi-squared test (categorical variables) and one-way ANOVA (continuous variables) were used to compare the differences between the METH groups. The Least Significant Difference multiple-comparison test was used for the *post-hoc* comparisons, and paired *t*-tests were used to examine changes in the OXA concentrations and scale scores within 1 month. Pearson's correlation analysis was used to determine the association between withdrawal symptoms and OXA concentrations at baseline. A *p*-value of <0.05 was selected as a statistically significant threshold. IBM SPSS Statistics was used to conduct all analyses (version 26.0).

## Results

### Demographic and Clinical Characteristics

The age difference between the METH users and healthy controls was not significant (*p* > 0.05). The three groups of METH users showed no significant differences in age, BMI, marital status, smoking habit, work status, educational attainment, or length of METH usage (*p* > 0.05). During the trial, nearly all METH users were given antipsychotics and mood stabilizers (see Table 1).

**Table 1:** Clinical characteristics of METH users and healthy controls.

**Clinical characteristics**	**Healthy control group** ** (*N* = 35)**	**Group 1** ** (*N* = 23)**	**Group 2** ** (*N* = 38)**	**Group 3** ** (*N* = 15)**	**Different test** ** (*p*-value)**
Age (years), M ± SD	37.43 ± 9.18	35.22 ± 9.69	37.84 ± 8.16	33.87 ± 7.19	0.375
BMI (kg/m^2^), M ± SD	–	23.01 ± 3.88	25.23 ± 3.49	24.29 ± 3.67	0.137
Marital status (married: *n*, %)	–	15 (65.20)	28 (75.68)	9 (69.20)	0.518
Smoking habit, *n* (%)	–	22 (95.60)	34 (91.89)	11 (84.60)	0.516
Employment status (employed: *n*, %)	–	17 (73.90)	23 (63.20)	8 (61.50)	0.569
Education, ≥college degree, *n* (%)	–	6 (26.00)	9 (24.40)	4 (30.70)	0.900
Length of METH use (years), M ± SD	–	4.41 ± 3.53	3.50 ± 3.79	6.13 ± 4.08	0.099
Length of METH abstinence (days), M ± SD	–	3.08 ± 1.62	35.6 ± 21.93	326.0 ± 339.95	<0.001
Length of METH abstinence (days), median (range)	–	3 (1–6)	30 (9–85)	180 (90–1,080)	
**Meditation treatment**, ***n*** **(%)**					
Antipsychotics		2 (8.70)	4 (10.53)	1 (6.67)	0.837
Mood stabilizer		2 (8.70)	2 (5.26)	1 (6.67)	
Antipsychotics + mood stabilizer		16 (69.56)	29 (76.30)	12 (80.00)	
Others		3 (13.04)	3 (7.89)	1 (6.67)	

*BMI, body mass index; Antipsychotics included olanzapine and risperidone; Mood stabilizers included valproate; Others included sedative-hypnotics and antidepressant; P-values were calculated based on one-way ANOVA (continuous data) and chi-squared test (categorical data); p = level of significance, <0.05*.

### OXA Measures

**Figure 1:**
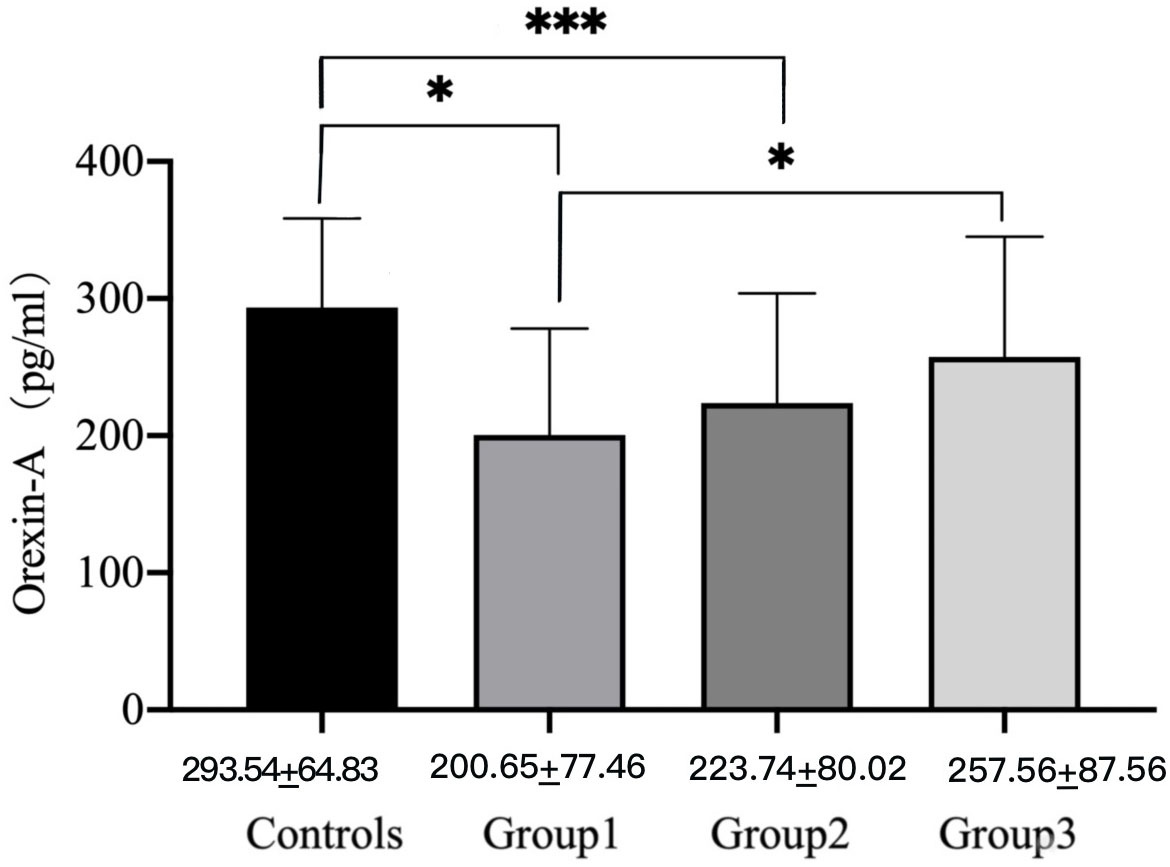
Orexin-A levels of male METH users in groups 1, 2, and 3 and the healthy control group on day 1. *P*-values were calculated using one-way ANOVA, *p* = level of significance, *p < 0.05, ***p < 0.001.

**Figure 2:**
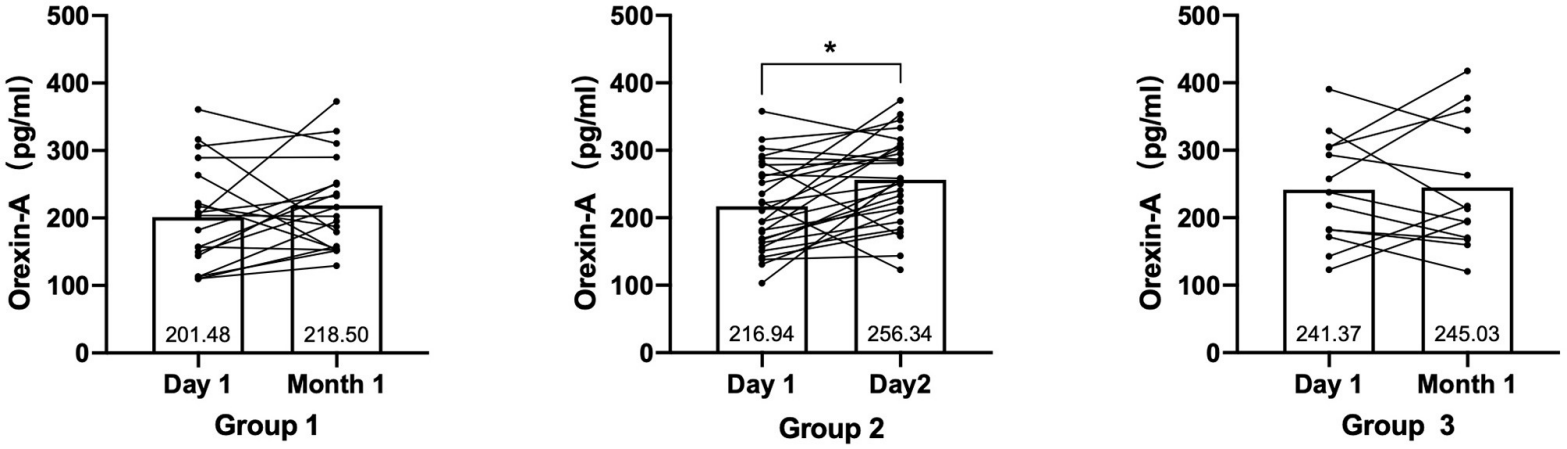
The change of Orexin-A levels in groups 1, 2, and 3 from day 1 to month 1. *P*-values for paired *t*-test, *p* = level of significance, *p < 0.05.

At baseline, groups 1 (200.65 ± 77.64 pg/ml, *P* < 0.001) and 2 (223.74 ± 80.02 pg/ml, *P* < 0.001) had significantly lower OXA concentrations than the healthy control group (293.54 ± 64.83 pg/ml), while group 3 (257.56 ± 87.65 pg/ml, *p* > 0.05) had no significant difference. At baseline, the concentrations of OXA in groups 1 (200.65 ± 77.64 pg/ml) and 3 (257.56 ± 87.65 pg/ml) were significantly different (*p* < 0.05) (see Figure 1). Furthermore, the OXA concentrations in group 2 increased considerably (*p* < 0.05) from day 1 (216.93 ± 81.04 pg/ml) of month 1 (256.33 ± 65.08 pg/ml), but neither group 1 nor 3 showed a significant increase (see Figure 2).

### Assessment of Depression and Anxiety

In our study, the 14-item Hamilton Anxiety Scale and 17-item Hamilton Depression Scale scores of the three groups reduced considerably from baseline to 1 month later (*p* < 0.05). Craving for METH in groups 1 and 2 reduced substantially within 1 month of study, and the improvement in sleep quality was found only in group 1 (see Table 2). We also investigated the relationship between METH withdrawal symptoms and the baseline OXA concentrations. In group 1, we discovered that the OXA concentrations were associated with anxiety (*r* = 0.587, *p* < 0.01) and depression (*r* = 0.664, *p* < 0.01) symptoms (see Table 3). However, in both groups 2 and 3, we failed to observe a significant association between the OXA concentration and such markers.

**Table 2:** Differences in Orexin-A concentration and withdrawal-related symptoms from day 1 to month 1.

	**Day 1**	**Month 1**	***T*-value (df), *p*-value**
**Orexin-A (pg/ml)**			
Group 1	201.48 ± 76.22	218.50 ± 68.46	−0.988 (18), 0.336
Group 2	216.93 ± 81.04	256.33 ± 65.08	−2.336 (26), 0.020^*^
Group 3	241.36 ± 80.12	245.03 ± 95.34	−0.178 (12), 0.862
**HAMA scores**			
Group 1	10.68 ± 3.69	7.00 ± 2.70	5.419 (18), *p* < 0.001^***^
Group 2	7.44 ± 3.69	6.00 ± 2.83	2.457 (26), 0.021^*^
Group 3	8.46 ± 3.04	4.38 ± 1.93	8.617 (12), *p* < 0.001^***^
**HAMD scores**			
Group 1	13.05 ± 3.34	9.65 ± 2.83	4.058 (18), 0.001^**^
Group 2	10.89 ± 4.48	8.78 ± 3.85	2.89 (26), 0.008^**^
Group 3	9.38 ± 2.81	6.07 ± 1.81	5.215 (12), *p* < 0.001^***^
**VAS scores**			
Group 1	4.00 ± 1.33	2.21 ± 1.32	4.232 (18), *p* < 0.001^***^
Group 2	2.62 ± 1.41	1.48 ± 1.71	4.087 (26), *p* < 0.001^***^
Group 3	1.13 ± 1.02	0.62 ± 0.87	1.328 (12), 0.209
**PSQI scores**			
Group 1	9.63 ± 4.09	6.58 ± 2.53	3.508 (18), 0.003^**^
Group 2	6.70 ± 2.75	5.85 ± 3.20	2.89 (26), 0.247
Group 3	7.15 ± 2.99	6.07 ± 1.93	1.815 (12), 0.095

*HAMA, hamilton anxiety scale; HAMA, hamilton depression scale; VAS, visual analog scale; PSQI, pittsburgh sleep quality index; Values are given as mean ± standard deviation. P-values were calculated based on the paired t-test, df, degrees of freedom. P = level of significance*,

* *p < 0.05*,

** *p < 0.01*,

*** *p < 0.001*.

**Table 3:** Relationship between orexin-A and METH withdrawal-related symptoms on day 1.

	**Orexin-A**	
	***r***	***p***
**Group 1**		
HAMA scores	0.587^**^	0.003
HAMD scores	0.664^**^	0.001
VAS scores	0.052	0.815
PSQI scores	0.179	0.413
**Group 2**		
HAMA scores	−0.126	0.411
HAMD scores	0.058	0.708
VAS scores	−0.033	0.83
PSQI scores	−0.053	0.731
**Group 3**		
HAMA scores	0.089	0.733
HAMD scores	0.177	0.635
VAS scores	−0.119	0.743
PSQI scores	−0.615	0.078

*HAMA, hamilton anxiety scale; HAMA, hamilton depression scale; VAS, visual analog scale; PSQI, pittsburgh sleep quality index; r, Pearson correlation coefficient; p = level of significance*,

*p < 0.01.

## Discussion

The aim of this study was to examine the plasma OXA concentrations, as well as their associations with METH withdrawal symptoms during abstinence. The concentration of OXA was considerably lower during early abstinence, and it progressively increased to near-normal. The decrease in OXA concentrations after the initial detoxification is only transitory and returns to normal over time. This change in OXA concentrations among METH users has not been observed in previous studies, to the best of our knowledge. Furthermore, during the initial abstinence, we discovered a substantial positive connection between the plasma OXA concentrations and negative emotion, which was not evident with prolonged abstinence. The findings suggested that the concentrations of OXA in the blood could predicted the severity of negative emotions experienced after early METH discontinuation.

Preclinical research suggests that METH actives orexin neurons in the hypothalamus (14), implicating orexin as a potential mediator of METH reinforcement. Consistent findings have also been obtained with cocaine (22, 23), alcohol (24), and opioids (25, 26). However, so far, it has not been determined how rewarding substances exposure regulates the transmission of orexin neurons. We found that chronic METH administration was associated with downregulation rather than elevation of OXA concentrations at baseline, while OXA concentrations were progressively increased during METH withdrawal. Previous studies suggest chronic cocaine intake in SD rats resulted in significantly decreased orexin mRNA levels (27). A similar reduction was also observed in chronic ethanol consumption, which is considered to be due to negative feedback by monoaminergic projections to orexin neurons (15). Although the stimulatory factors of orexin levels' changes are not yet fully clear, it is possible that chronic exposure to drugs of abuse led to reduced orexin expression, which may act as a protective mechanism against drug abuse. Alternatively, recovery of OXA levels during withdrawal may act as a compensation to balance orexin peptide loss.

Several previous studies have shown evidence of a decrease in orexin concentrations following drug METH withdrawal. One observational study reported that OXA concentrations of METH users decreased during the initial phase of withdrawal, and the pattern for more than 2 weeks (18). Another research found that METH and opiate users had lower orexin, as well as OXA, during withdrawal than healthy controls (17). The decrease in orexin was also reflected in patterns observed during the withdrawal stages of other drugs. Long-term ketamine users had a lower OXA concentration during the early stages of withdrawal (28). Based on previous studies, our findings suggested that the OXA concentrations may be reduced during early METH abstinence and eventually rebound after METH abstinence.

Orexin also acts as a critical modulator in the expression of drug withdrawal symptoms, according to an increasing body of research (29, 30). A clinical study involving alcohol-dependent individuals discovered a strong, positive correlation between the orexin concentrations and depressive symptoms during acute alcohol withdrawal (31). Consistent with this, our results also indicate a significant positive association between the OXA concentrations and negative emotions during initial METH withdrawal. Li et al. al also reported that rats showed reduced orexin concentrations during 60 days of morphine withdrawal, which is consistent with the anhedonia induced by prolonged withdrawal (32), further suggesting a close relationship between the processes. In summary, there is still no consensus on how orexin signaling is accountable for depressive-like behavior, however, the downregulated orexin concentration may act as a risk factor for negative emotion during the initial METH withdrawal.

METH-dependent individuals are reported to experience drowsiness and decreased appetite during the initial phase of withdrawal lasting for ~7–10 days, after which their appetite recovers and wakefulness is gradually promoted (3). Orexin is required for the stabilization of wakefulness, and its absence causes sleep-wake instability. We discovered a considerable improvement in sleep quality among METH users during a 1-month study and noted a trend toward a negative relationship between orexin levels and PSQI scores in Groups 2 and 3. In consistent, a preclinical study showed that orexin expression was considerably downregulated in alcohol-dependent mice during acute withdrawal, which can be associated with the mechanism of daytime sleepiness during alcohol withdrawal (33). In summary, these results supported the hypothesis that decreased OXA of METH users is linked to the deficits in sleep quality during initial withdrawal.

Craving, a red flag for relapse, is thought to facilitate continued drug-taking. Previous evidence indicates that cravings for METH decreased dramatically after 2 weeks of abstinence and remained low for the next 5 weeks (3). In our study, the cravings for METH in groups 1 and 2 were significantly more intense at baseline than in group 3, although they decreased to a near-minor and negligible concentration after a month. Our results also indicated that METH users had a decreased need for METH after several weeks of abstinence, consistent with prior evidence that craving abated with longer abstinence among METH users (34). However, what we cannot be ignored is that the VAS is limited by its subjective nature, which may influence the accuracy of our results. Combining the valued Impulsive Behavior Scale to assess the craving after cue exposure may be helpful for a more objective analysis.

Although a large amount of preclinical work indicates that increased orexin activity is linked to drug-seeking behaviors (23, 35), we found METH users with reduced orexin levels during initial abstinence exhibited high cravings. Orexin has been demonstrated to be able to regulate the mesolimbic DA neuronal activity and synaptic plasticity (36, 37), thus, the reduced orexin level may contribute to decreased dopamine response to METH during initial withdrawal. The impairments in dopamine neurotransmission can promote persistent drug use in METH-dependent individuals (38), similar results have been observed among cocaine addicts (39). In addition, our results demonstrate that craving scores decrease with time, but orexin levels increase during abstinence. Similarly, a strong negative association between circulating orexin levels and nicotine demand has been discovered, and the orexin concentration has been proposed as a prospective marker and risk factor for smoking relapse (40, 41). These results may contribute to better understanding of the relationship between decreased orexin and high cravings during the early stages of withdrawal.

While these findings are encouraging, there are some limitations to this study that need to be addressed in future research. All METH users were treated with minor dosages of antipsychotics and mood stabilizers during the research. Nearly all methamphetamine users (91.6%) received therapy, with 77.49 percent receiving a combination of antipsychotics and mood stabilizers. OXA concentration was found to be increased in schizophrenia patients treated with antipsychotics, which was linked to a decreased incidence of metabolic syndrome (42). A similar increase in OXA protein concentration in LH was observed in rats treated with long-term use of antipsychotics (43). Further, the medication treatment also affects METH withdrawal symptoms, particularly negative emotions and sleep quality, which makes some of the effects difficult to interpret. To exclude the complex effects of medications on symptom assessment and plasma OXA concentration measurement in the future, it is necessary to control the amount of medication or recruit METH users who are not treated. Furthermore, there are also significant sex disparities in orexin expression (44), and prior research demonstrates that female orexin may be expressed at higher levels in the central nervous system (45). Therefore, future studies should include female METH users for further comparison. Finally, the sample size of this study was small, and the three groups did not have equal distributions of participants, necessitating a larger sample size in future investigations.

Overall, the current study found a significant reduction in OXA concentrations during the early stages of METH withdrawal, as well as recovery with continued abstinence. Furthermore, during the early stages of METH withdrawal, we discovered an association between OXA and abstinence-related negative emotions. Orexins serve as potentially significant signals in the neural circuit during METH dependence and withdrawal, which may promote the development of new relapse preventive strategies. We may better comprehend the neurobiological process behind METH addiction if we have a better understanding of the changes in the trend of OXA concentrations. As a result, the orexin system can be a key target for METH dependence pharmacotherapies.

## Data Availability Statement

The raw data supporting the conclusions of this article will be made available by the authors, without undue reservation.

## Ethics Statement

The studies involving human participants were reviewed and approved by Clinical Center Ethics Committee of the Xiangya Second Hospital of Central South University, China. The patients/participants provided their written informed consent to participate in this study.

## Author Contributions

XX and LG attributed the concept and design of the study and completed the data analysis and manuscript. LG, AH, and XZ obtained and acquired the data. All authors reviewed and approved the final manuscript.

## Funding

The work was supported by grants from the Natural Science of Hunan Province, China (Grant No. 2021JJ30962) to XX, the Fundamental Research Founds for the Central Universities of Central South University (Grant No. 2021zzts1058), and the National Natural Science of China (Grant No. 81571306) to XX.

## Conflict of Interest

The authors declare that the research was conducted in the absence of any commercial or financial relationships that could be construed as a potential conflict of interest.

## Publisher's Note

All claims expressed in this article are solely those of the authors and do not necessarily represent those of their affiliated organizations, or those of the publisher, the editors and the reviewers. Any product that may be evaluated in this article, or claim that may be made by its manufacturer, is not guaranteed or endorsed by the publisher.
